# *VviRafS5* Is a Raffinose Synthase Involved in Cold Acclimation in Grapevine Woody Tissues

**DOI:** 10.3389/fpls.2021.754537

**Published:** 2022-02-15

**Authors:** Henrique Noronha, Angélica Silva, Tiago Silva, Sarah Frusciante, Gianfranco Diretto, Hernâni Gerós

**Affiliations:** ^1^Department of Biology, Centre of Molecular and Environmental Biology (CBMA), University of Minho, Braga, Portugal; ^2^Centre for the Research and Technology of Agro-Environmental and Biological Sciences (CITAB), University of Trás-os-Montes e Alto Douro, Vila Real, Portugal; ^3^Casaccia Research Center, ENEA, Italian National Agency for New Technologies, Energy and Sustainable Economic Development, Rome, Italy; ^4^Department of Engineering, Centre of Biological Engineering (CEB), University of Minho, Braga, Portugal

**Keywords:** *Vitis vinifera*, raffinose biosynthesis, RFOs sugars, cold acclimation, woody tissues

## Abstract

The accumulation of raffinose family oligosaccharides (RFOs) is a hallmark of plant response to different abiotic stresses, including cold. The synthesis of galactinol, by galactinol synthases (GolS), and raffinose, by raffinose synthases (RafS), are fundamental for stress-induced accumulation of RFOs, but the role of these enzymes in the cold response of grapevine (*Vitis vinifera* L.) woody tissues is still unclear. To address this gap in the literature, 1-year-lignified grapevine canes were incubated at 4°C for 7 and 14 days and tissues were analyzed for sugar content and gene expression. Results showed that, in parallel to starch breakdown, there was an increase in soluble sugars, including sucrose, glucose, fructose, raffinose, and stachyose. Remarkably, abscisic acid (ABA) levels increased during cold acclimation, which correlated with the increased expression of the key ABA-synthesis genes *VviNCED2* and *VviNCED3*. Expression analysis of the *VviGolS* and *VviRafS* family allowed the identification of *VviRafS5* as a key player in grapevine cold response. The overexpression of *VviRafS5* in *Saccharomyces cerevisiae* allowed the biochemical characterization of the encoded protein as a raffinose synthase with a size of ~87 kDa. In grapevine cultured cells, *VviRafS5* was upregulated by cold and ABA but not by heat and salt stresses. Our results suggest that ABA accumulation in woody tissues during cold acclimation upregulates *VivRafS5* leading to raffinose synthesis.

## Introduction

Among different environmental conditions, low temperature (0–15°C) is the one that mostly affects the geographic distribution of plants, productivity, and yield quality (Thomashow, 1999; Yamaguchi-Shinozaki and Shinozaki, 2006). This is the case of grapevine (*Vitis vinifera* L.) that grows primarily in temperate and subtropical regions, whose productivity may be severely affected by environmental fluctuations (Bock et al., 2013). In particular, cold stress, such as rapid temperature drop in late fall, freezing temperatures in midwinter, or early spring frost, can seriously damage grapevines by reducing their growth and photosynthesis, retarding flowering, and compromising fruit production (Fennell, 2004; Hendrickson et al., 2004; Ait Barka et al., 2006; Xin et al., 2013). Also, when exposed to harsh winters, grapevine may suffer from delayed and unsynchronized budbreak, dieback of perennial organs, or even plant death (Antivilo et al., 2020).

Cold acclimation is a physiological process leading to freezing tolerance in plants, which is critical for their ability to withstand harsh winter conditions (Thomashow, 1999; Theocharis et al., 2012). A set of biochemical and molecular adjustments contribute to the plant’s adaptation during cold stress, including reduced water uptake, accumulation of cytosolic Ca^2+^, increased levels of Reactive Oxygen Species (ROS) and the activation of ROS scavenging systems, induction of cold-specific genes (e.g., CBF, C-repeat Binding Factor and ICE genes, Inducer of CBF Expression) and synthesis of cold-related proteins (e.g., CSDPs, cold shock domain proteins), and accumulation of abscisic acid (ABA) and osmolytes (Thomashow, 1999; Mahajan and Tuteja, 2005; Theocharis et al., 2012). Among these, sugar accumulation is one of the most documented plant responses to low temperature (Kaplan et al., 2004; Dietze et al., 2014). In particular, the accumulation of raffinose family oligosaccharides (RFOs), which are α-1,6-galactosyl extensions of sucrose (Supplementary Figure 1), is fundamental during cold hardiness (Grant et al., 2009; Egert et al., 2013). Although the protective role of RFOs still remains unclear, they have been associated with the scavenging of ROS, as functioning as osmoprotectants, stabilizing biological membranes, and protection of photosystem II (Taji et al., 2002; Li et al., 2020).

The first step in RFOs formation is catalyzed by galactinol synthase (GolS, EC 2.4.1.123), which forms galactinol from myo-inositol and UDP-galactose. Subsequently, raffinose synthase (RafS; EC 2.4.1.82) catalyzes the synthesis of raffinose by transferring the galactosyl unit from galactinol to sucrose, yielding a trisaccharide (sucrose-galactose). In some species, further elongations may be catalyzed by galactan:galactan galactosyl transferases (GGTs) yielding stachyose (tetrasaccharide) and verbascose (pentasaccharide; Supplementary Figure 1; Peterbauer et al., 2002; Egert et al., 2013; Sengupta et al., 2015).

GolS is considered a key regulator of RFO synthesis and accumulation (Vinson et al., 2020), and the overexpression of two wheat *GolS* (*TaGolS1* and *TaGolS2*) in rice promotes the accumulation of galactinol and raffinose and increased cold-stress tolerance (Shimozaka and Ozawa, 2015). Also, the expression of a *GolS* from the desert plant *Ammopiptanthus nanus* (*AnGolS1*) in tomato improved its tolerance to cold stress (Liu et al., 2020). Interestingly, it has been shown that the *Vitis amurensis AQUILO* transcription factor improves cold response of *Arabidopsis thaliana* transgenic plants by regulating *AtGolS1/3* and *AtRafS5* and increasing RFO content (Sun et al., 2018). Recently, *ZmRAFS* has been associated with increased plant tolerance to drought (Li et al., 2020), and also with increased raffinose content and enhanced chilling tolerance after overexpression of the *ZmDREB1A* transcription factor (Han et al., 2020). Nonetheless, few studies explored the role of GolS and RafS during cold acclimation in woody tissues.

Although ABA is recognized as a central player in abiotic stress responses (Vishwakarma et al., 2017), like drought and cold, its relation with RFO metabolism is still limited. Interestingly, it was shown that a *GolS* from *Boea hygrometrica* (*BhGolS1*) is induced by dehydration and ABA (Wang et al., 2009) and that alfalfa somatic embryos treated with ABA show an increased expression of GolS and accumulation of galactinol, raffinose, and stachyose (Blöchl et al., 2005).

Reports have shown that grapevine synthesizes proline and phenolic compounds and accumulates starch and soluble sugars to overcome cold stress (Ait Barka et al., 2006; Fernandez et al., 2012), but there is still a gap of knowledge concerning the role of RFOs in plant adaptation to cold. In grapevine, *VviGolS1* was associated with the response to heat stress by synthesizing galactinol, although the accumulation of raffinose or stachyose was not detected (Pillet et al., 2012). Also, different *GolSs* are expressed in *V. amurensis* and *V. vinifera* plantlets in response to cold stress (Chai et al., 2019). In this work, 1-year-lignified canes from an important Portuguese regional variety—Vinhão—were harvested at the beginning of autumn and incubated at 4°C to simulate cold acclimation. Starch, soluble sugars (sucrose, glucose, fructose, raffinose, and stachyose), and ABA were then quantified after 7 and 14 days of acclimation. Expression analysis of *VviGolS* and *VviRafS* families was carried out, which allowed the identification of *VviRafS5* as a key player in grapevine cold response in woody tissues. The overexpression of *VviRafS5* in yeast enabled the *in vitro* biochemical characterization of the enzyme in protein extracts. Notably, *VviRafS5* is upregulated by cold and ABA but not by heat and salt stresses as observed in grapevine cell suspensions.

## Materials and Methods

### Plant Material

**Figure 1:**
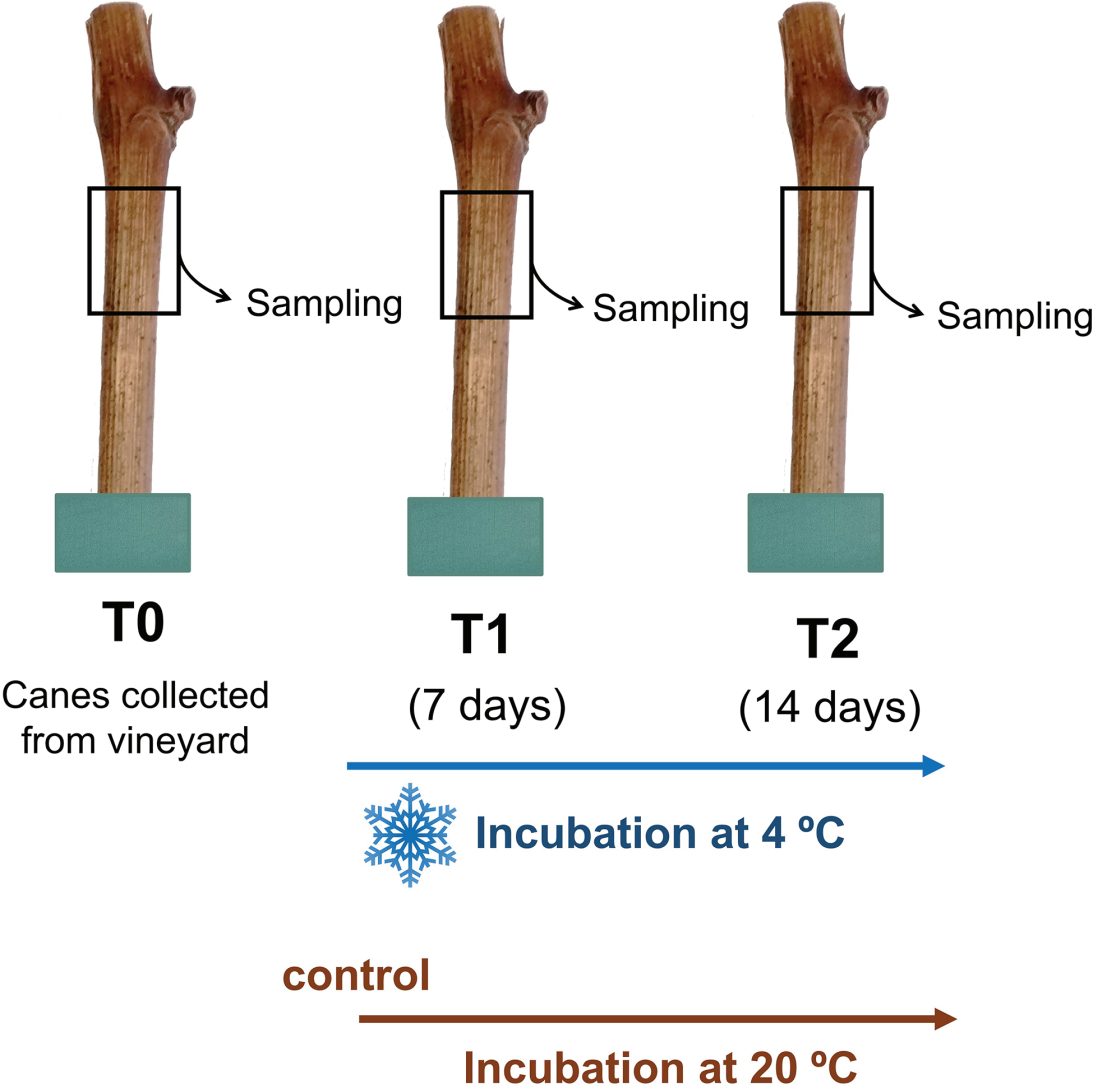
Experimental design. *Vitis vinifera* woody canes (cv. Vinhão) cut into single bud segments were incubated in at 4°C for 7 and 14 days (T1 and T2, respectively). Samples at the beginning of the experiment were also collected (T0). Control canes were also incubated at 20°C for 7 and 14 days.

Field-grown grapevine (*V. vinifera* L.) canes were collected after fruit harvest from a commercial vineyard of the Controlled Appellation (DOC) region of Vinhos Verdes in the northwest region of Portugal (41°48′45.3″N 8°24′36.4″W) in 2019. Lignified shoots of similar diameter (~2 cm) were cut into single bud segments of approximately 15 cm, its basal ends were inserted into wet floral foam, and the top ends were sealed with paraffin film to prevent dehydration, as previously reported (Noronha et al., 2021). Samples were then incubated at 4°C for 7 (T1) and 14 days (T2). Shoots not subjected to cold were used immediately after their collection (T0; Figure 1). Control canes were collected in the same conditions, during 2021, and incubated for the same periods at room temperature (20°C). At each incubation period, three biological replicates (four similar sized canes per replicate) were sampled. The periderm of each sample was removed with a scalpel, frozen with liquid N_2_ to be ground in a IKA A11 basic analytical mill and stored at −80°C. Part of these samples was freeze-dried in a Christ-Alpha 2–4 LD lyophilizer for quantification of raffinose, stachyose, and starch.

Gamay Fréaux cultured cells were grown in liquid Gamborg B5 supplemented with 2% (w/v) sucrose and .025% (w/v) casein hydrolysate and maintained in 250 ml flasks on a rotatory shaker at 100 rpm and 24°C. Cells were subcultured weekly by transferring 10 ml aliquots into 40 ml of fresh medium. To study the effect of different temperatures on *VviRafs5* expression, 20 ml aliquots of 6-days-old cells were incubated for 24 h at 24 (control), 4, and 37°C. The effect of 100 mm NaCl and 100 μm ABA was tested in cells cultivated for 24 h at 24°C. Cells were collected by vacuum filtration, ground in liquid N_2_, and stored at −80°C.

### Sugar Quantification by High Performance Liquid Chromatography

The extraction of sugars was performed as reported by Conde et al. (2015) adapted from Eyéghé-Bickong et al. (2012). In short, for sucrose, glucose, and fructose quantification, 150 mg of fresh weight (FW) cane tissue was mixed with 1 ml of ultrapure H_2_O, vortexed, and mixed with 1 ml of chloroform. The same procedure was done with lyophilised material for raffinose and stachyose quantification. The biphasic solvent was vortexed for 5 min and incubated at 50°C for 30 min with continuous shaking (150 rpm). Samples were then centrifuged at 15000 × *g* for 10 min and the supernatant was collected and filtered with a .45 μm nitrocellulose filter. Sugars were quantified by HPLC-RI with a Rezex RCM–Monosaccharide Ca^2+^ (8%) column (Phenomenex) at a flow rate of .6 ml min^−1^ at 40°C using water as the mobile phase. Twenty μl of each sample were injected and sugar concentration was measured after the comparison of the peak area and retention time with calibration curves.

### Starch Quantification

Starch was quantified in canes according to Silva et al. (2017). To remove soluble sugars, lyophilized cane tissues (50 mg DW) were extracted three times with 5 ml 80% ethanol, and starch grains were gelatinized by autoclaving. Starch was enzymatically degraded by α-amylase (AMY; 1 U, Sigma–Aldrich) and β-glucosidase (10 U, Sigma–Aldrich) in a medium containing 200 mm sodium acetate (pH 5.5) and released sugars were measured with the DNS method.

### *In silico* Studies

To identify grapevine RFO gene family members, *Arabidopsis thaliana* sequences of GolS and RafS were blasted against the genome of *V. vinifera* L. (12X) using the online platform Phytozome.^1^ Annotated genes were named following phylogenetic analysis using amino acid sequences from *V. vinifera* and *Arabidopsis thaliana* obtained from the National Center of Biotechnology (NCBI) and Uniprot (Supplementary Figure 2). Sequence alignment was performed with PRANKSTER (Whelan and Goldman, 2001) and Genedoc (Nicholas et al., 1997). The phylogenetic tree was constructed using these alignments with PROTDIST, NEIGHBOR, and RETREE from the PHYLIP software package (Felsenstein, 1989) and Mega 4 (Tamura et al., 2007).

### RNA Isolation and cDNA Synthesis

Total RNA from cane tissues and cultured cells was isolated as previously described (Reid et al., 2006) with some adaptations. For each condition, 500 mg of frozen tissue was mixed with 1 ml of extraction buffer containing 300 mm Tris HCl (pH 8.0), 25 mm EDTA, 2.0 M NaCl, 2% CTAB, 2% PVP (K-30), and 30 mm DTT. Samples were then incubated at 60°C for 15 min and shaken every 5 min. Then, mixtures were extracted twice with 850 μl of chloroform: isoamyl alcohol (24:1) followed by centrifugation at 15000 × *g* for 15 min at 4°C. Subsequently, .1 vol of 3 M NaOAc (pH 5.2) and .6 vol of isopropanol were added to the aqueous phase, followed by incubation at −80°C for 1 h. The mixture was centrifuged at 15000 × *g* at 4°C during 30 min and later resuspended in 100 μl of ultrapure H_2_O. To purify the samples, the GRS Total RNA Kit—Plant (GriSP, Lda.) was used following the manufacturer’s instructions. RNA concentration and purity were quantified spectrophotometrically in the NanoDrop ND-1000 (Thermo Fisher Scientific Inc.) and integrity checked in a 1% (w/v) agarose gel. First strand cDNA synthesis was performed using Xpert cDNA Synthesis Mastermix protocol (GriSP, Lda.), according to the manufacturer’s instructions.

### Real-Time PCR Studies

Quantitative real-time PCRs were performed with Xpert Fast SYBR Blue (GriSP, Lda.) along with the conditions previously optimized in a CBX96 Real-Time Detection System (Bio-Rad). The amplification protocol included an initial denaturation step at 95°C for 3 min, followed by additional 40 cycles of denaturation for 3 s at 95°C, annealing for 20 s at 55°C, and 20 s at 72°C. Experiments were done in biological replicates and then interpreted with the software Bio-Rad CFX Manager (Bio-Rad), while *VviGAPDH* (glyceraldehyde-3-phosphate dehydrogenase) and *VviACT1* were used as internal control. The primers used in this study are listed in Supplementary Table 1.

### Cloning of *VviRafs5* and Its Overexpression in Yeast Cells

Due to experimental difficulties in using bacterial hosts, gap repair was used to clone *VviRafs5* into the *pYES-DEST52* vector (V5-epitope; Invitrogen) using *Saccharomyces cerevisiae* INVSc1 (Bessa et al., 2012). *VviRafS5* CDS, without the stop codon (allowing the production of a *VviRafS5*-V5-epitope fusion protein), was amplified using primers listed on Supplementary Table 1 (*VviRafS5*-Gap-FW and *VviRafS5*-Gap-RV). Fragment amplification was done using NZYTaq II DNA polymerase (NZYTech, Lda), according to the manufacturer’s instructions. pYES-DEST52 (Invitrogen) vector was digested with EcoRI (Thermo Scientific) and SmaI (Thermo Scientific) and purified using GRISP PCR & Gel Band Purification (GriSP, Lda.). The transformation of INVSc1 yeast was carried out using the LiAc/SS-DNA/PEG method and linearized pYES-DEST52 and *VviRafS5* PCR product (Gietz and Woods, 2002). As control, yeast cells were also transformed with the empty vector. Transformants were selected on solid synthetic medium: .7% YNB (Alfa Aesar), 1.3% dropout (US Biological; without uracil), .5% ammonium sulfate, 2% glucose, and 2% agar. Positive clones, confirmed by PCR, were cultivated overnight at 30°C in liquid synthetic medium with 2% (w/v) glucose and protein synthesis was induced by diluting cells to DO_640nm_ .4 in synthetic medium supplemented with 2% galactose and allowed to grow overnight at 30°C. Cells were then collected, washed twice with deionized water, and resuspended in extraction buffer (pH 7.5, 50 mm HEPES, 25 mm NaCl, 1 mm PMSF, 1 mm DTT, and 1 mm EDTA). Acid-washed glass beads were added and cell lysis was accomplished in the cell disruptor (Scientific) with 3 cycles of 30 s homogenization followed by 1 min on ice. The mixture was centrifuged at 16000 × *g* for 20 min at 4°C, the supernatant was collected, and sugars present in the crude protein extracts were removed using a Amicon Ultra-4 10,000 NMWL (Merck), following the manufacturer’s instructions. Protein extracts were used to study *VviRafS5* activity.

### Functional Characterization of VviRafs5

Protein concentration was assessed with the Bradford assay. To functionally characterize *VviRafS5* protein, galactinol and sucrose were used as substrates in saturating concentrations according to Li et al. (2020). The reaction was conducted in a 200 μl reaction system containing reaction buffer (50 mm HEPES; pH 7.5), 30 mm sucrose, 25 mm galactinol, and 50 μl of crude yeast extract. Reactions proceeded for 2 h at 37°C. Produced raffinose was measured by HPLC as described above.

### Western Blot Analysis

Protein samples obtained as described above were separated on 10% acrylamide gels as described by Laemmli (1970). Proteins were transferred to a nitrocellulose membrane during 1.5 h at 100 V and were blocked during 1 h in TRIS-buffered saline containing .1% (v/v) Tween-20 (TBS-T) with 5% (w/v) skimmed milk powder. The membranes were probed against V5-tag (Anti-V5 antibody, 1:3000 dilution, Sigma) during 1 h at room temperature in blocking solution, followed by an incubation with an anti-rabbit peroxidase-conjugated antibody (Sigma) at 1:10000 dilution in TBS-T, for 45 min. The immunodetection was accomplished with the chemiluminescent ECL detection substrate (Bio-Rad) and observed in the ChemiDoc system (Bio-Rad).

### ABA and ABA-Related Metabolites Quantification

ABA and ABA catabolites detection and quantification were performed as reported in Diretto et al. (2020) and Barja et al. (2021). Briefly, 50 mg freeze-dried grounded cane samples were extracted using unbuffered Tris-ethyl acetate as reported before (Welsch et al., 2008). LC-HRMS was carried out using an Ultimate UHPLC system with a photodiode array detector (Dionex), and a Q-exactive quadrupole Orbitrap mass spectrometry system (Thermo Fisher Scientific; LC-HRMS) equipped with an electrospray ionization (HESI) source, operating in negative ion mode, as previously described (Di Meo et al., 2019) with the following modifications: with nitrogen as sheath and auxiliary gas set at 35 and 25 units, respectively. The vaporizer and capillary temperatures were set at 280 and 320°C, respectively. The discharge current was set to 4.0 μA and S-lens RF level set at 50. Internal standard-based quantification was carried out using the MS data, while retention times and MS2 fragmentation patterns were used for ABA identification by using authentic reference standards (trans-ABA from OlChemIm and ()-ABA from Sigma, St. Louis, MO, United States).

### Statistical Analysis

The results were statistically analyzed by ANOVA tests (one-way and two-way ANOVA) using Prism vs. 7 (GraphPad Software, Inc.). Statistical differences between mean values are marked with letters for the different conditions or were marked with asterisks (^*^p ≤ .05; ^**^p < .01; ^***^p < .001; and ^****^p < .0001).

## Results

### Starch Decreases and Soluble Sugars Increase During Cold Acclimation of Grapevine Canes

**Figure 2:**
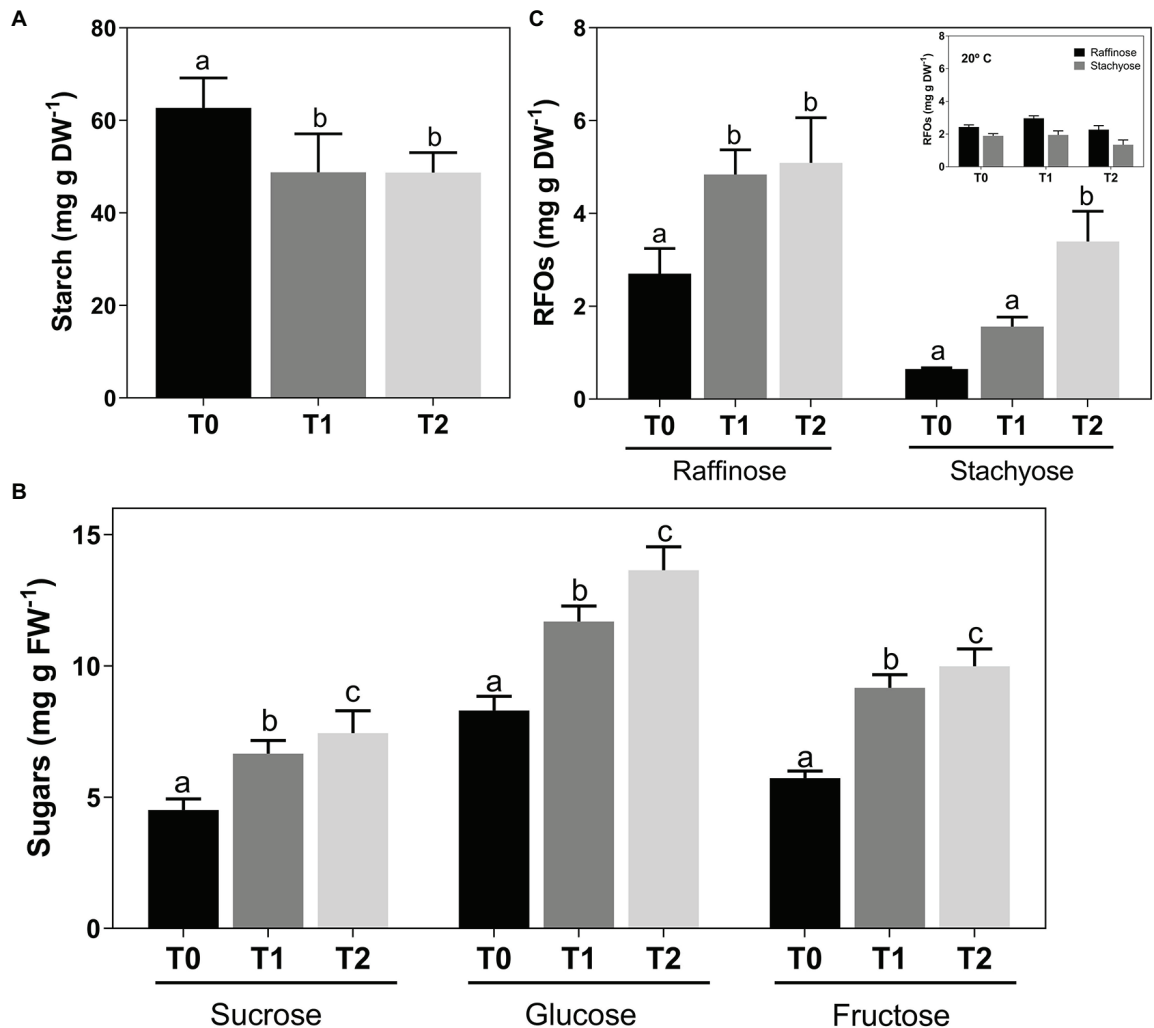
Concentration of sucrose, glucose, and fructose **(A)**, starch **(B)** and raffinose, and stachyose **(C)** in *Vitis vinifera* cv. Vinhão canes incubated at 4°C up to 14 days. Insert, raffinose, and stachyose levels in canes incubated at 20°C up to 14 days. Results indicate the mean ± SD of three biological replicates per condition. Different letters stand for significant differences (*p* ≤ .05).

Results showed a steadily reduction in the amount of stored starch in woody canes after cold treatment, from 62.74 ± 5.90 at T0 to 48.74 ± 3.87 mg g DW^−1^ at T2 (Figure 2A). In parallel to starch degradation, an increase in sucrose levels was observed, from 4.52 ± .40 to 7.44 ± .80 mg g FW^−1^ at T2 (Figure 2B). Glucose and fructose also increased from 8.31 ± .51 and 5.73 ± .26, respectively, at T0 to 13.65 ± .83 and 9.99 ± .62 mg g FW^−1^ at T2 (Figure 2A). Regarding RFOs, raffinose at T2 almost doubled its amount from T0, increasing from 2.70 ± .44 to 5.09 ± .80 mg g DW^−1^, while a 5-fold increase in stachyose was observed, from .65 ± .02 at T0 to 3.40 ± .53 mg g DW^−1^ T2 (Figure 2C). Canes incubated at room temperature showed no changes in raffinose and stachyose content (Figure 2, inset).

### ABA Is Involved in the Cold Response of Grapevine Canes

**Figure 3:**
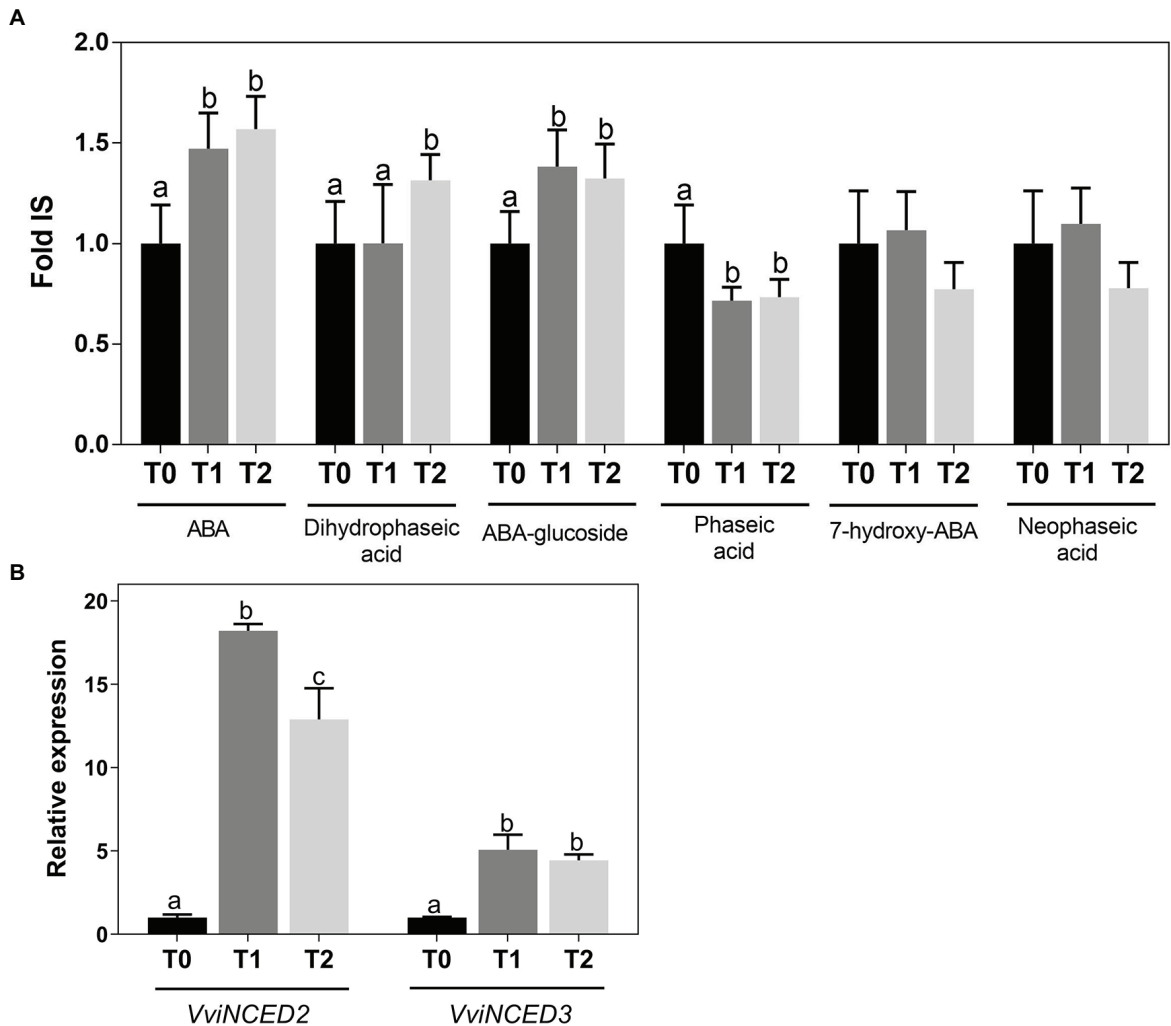
Quantification of ABA and ABA-related metabolites by LC-HRMS **(A)** and expression of *VviNCED* genes **(B)** in *Vitis vinifera* cv. Vinhão canes incubated at 4°C up to 14 days. Results indicate the mean ± SD of three biological replicates per condition. Different letters stand for significant differences (*p* ≤ .05).

ABA and ABA-related metabolites were quantified in grapevine woody tissues by LC-HRMS. Results showed that ABA increased from T0 to T2 together with ABA-glucoside and dihydrophaseic acid, while phaseic acid decreased (Figure 3A). Accordingly, the expression of *VviNCED2* and *VviNCED3*, which code for 9-*cis*-epoxycarotenoid dioxygenases and are considered major control points of ABA synthesis, was upregulated following cold treatment (Figure 3B).

### Cold Promotes a Transcriptional Modification of Key Genes Involved in RFOs Metabolism

**Figure 4:**
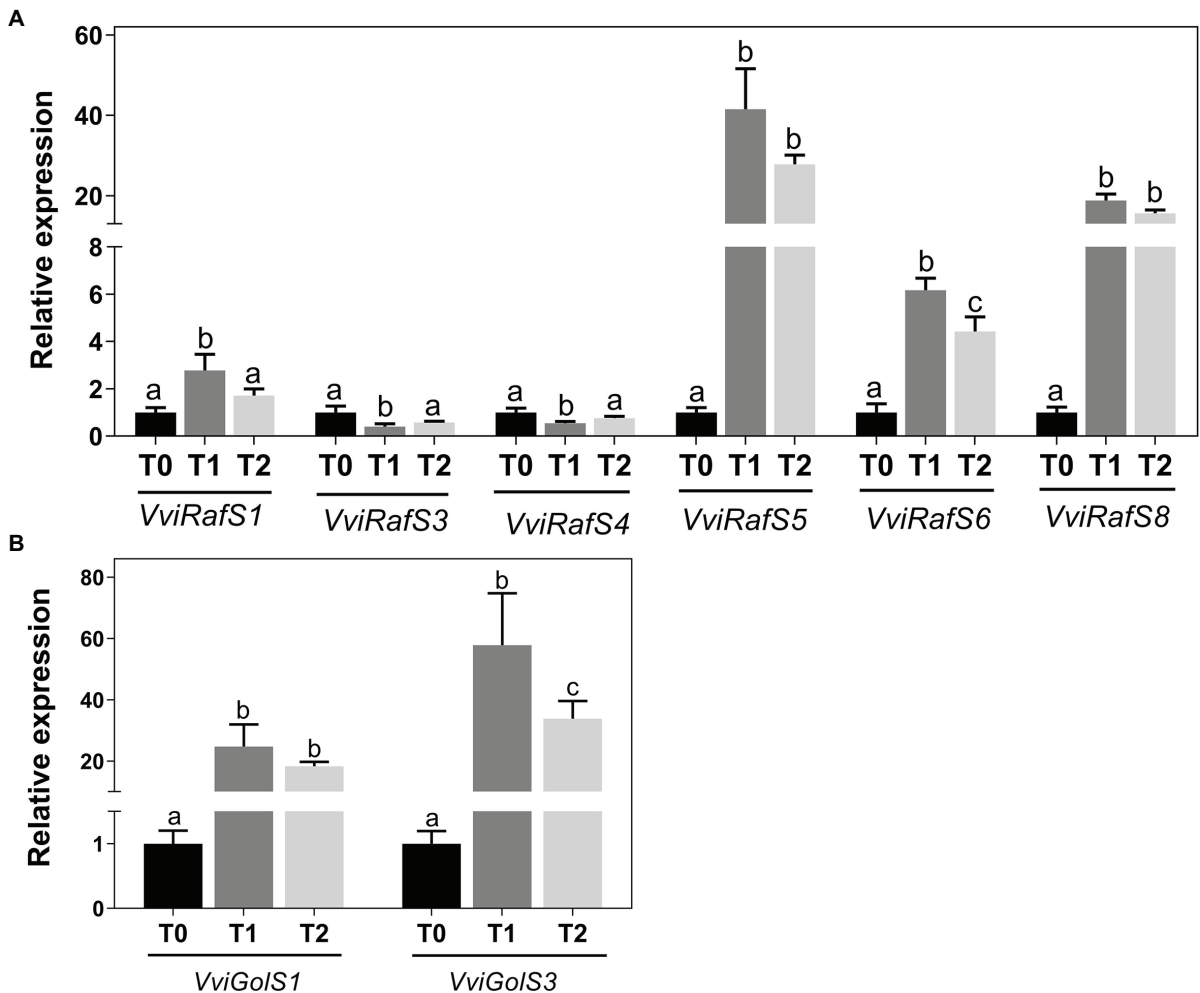
Transcriptional analysis of *VviGolSs* **(A)** and *VviRafSs* **(B)** in *Vitis vinifera* cv. Vinhão canes incubated at 4°C up to 14 days. Results indicate the mean ± SD of three biological replicates per condition. Different letters stand for significant differences (*p* ≤ .05).

Real-Time PCR analysis was performed to determine the expression of genes coding for enzymes responsible for the synthesis of RFOs during cold adaptation of grapevine woody tissues (Figure 4). Eight raffinose synthase genes, *VviRafS1-8*, and one stachyose synthase, *VviStaS1*, were identified (Supplementary Figure 3) and their expression in grapevine woody tissues following incubation at 4°C was analyzed (Figure 4B). *VviRafS1, −3*, and *− 4* were not transcriptionally modified in response to cold and *VviStaS1* and *VviRafS2* and *− 7* were not detected by qPCR in these tissues. On the contrary, the steady-state transcript levels of *VviRafS6* increased by up to 6-fold compared to T0 the expression of *VviRafS5* and *− 8* was strongly upregulated up to 40-fold. Furthermore, results showed that cold treatment strongly upregulated the expression of *VviGolS1* and *VviGolS3*, which are key enzymes in galactinol synthesis (Figure 4B).

### *VviRafS5* Codes for an Active Enzyme in Yeast Cells

**Figure 5:**
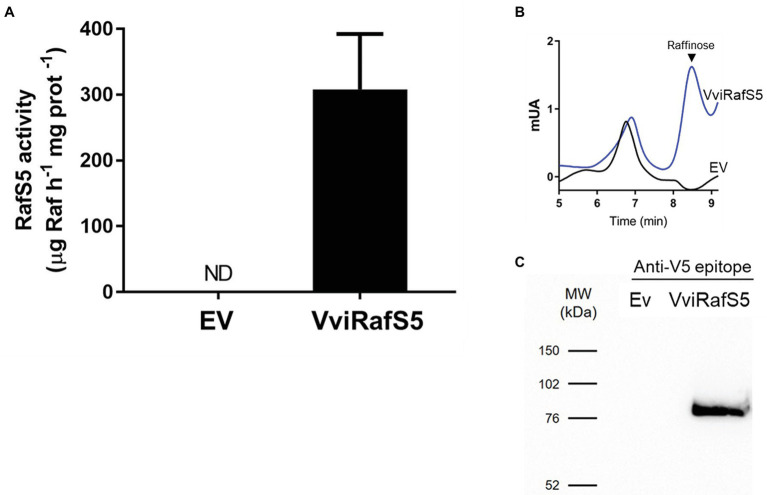
Functional characterization of *VviRafS5*. Production of raffinose in protein lysates from INVSc1 yeast expressing *VviRafS5*:V5-tag, or the empty vector (EV), when incubated with sucrose and galactinol **(A)**. Raffinose synthesis was determined by HPLC **(B)**. Protein production was confirmed trough Western blotting using anti-V5 antibody **(C)**.

*VviRafS5* was cloned into *S. cerevisiae* strain INVSc1 and its ability to synthesize raffinose *in vitro* was studied by HPLC (Figure 5). Results showed that yeast protein extracts containing *VviRafS5*-V5-tag are able to synthesize raffinose in the presence of saturating galactinol and sucrose (Figures 5A,B). Also, the presence of *VviRafS5*-V5-tag in yeast protein extracts was confirmed by Western blot using an anti-V5 antibody, with a molecular weight of ~87 kDa (Figure 5C).

### *VviRafS5* Transcriptional Regulation by Different Stresses

**Figure 6:**
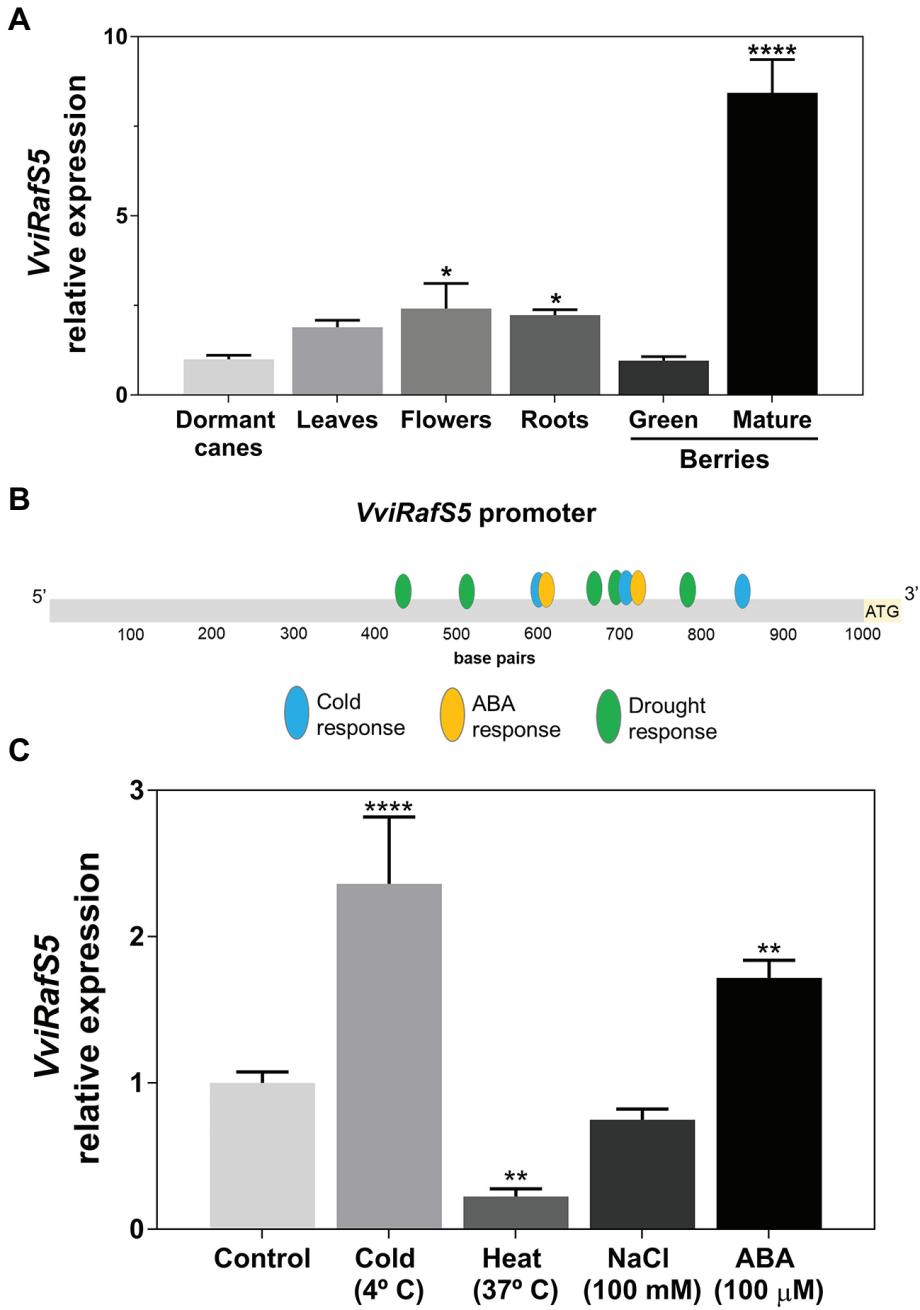
Transcriptional regulation of *VviRafS5*. *Cis*-regulatory elements found in the *VviRafS5* promotor involved in stress response **(A)**. Transcriptional analysis of *VviRafS5* in Gamay Fréaux cells after 24 h of cold (4°C), heat (37°C), and salt (100 mm) stresses and ABA (100 μm) elicitation **(B)**. Transcriptional analysis of *VviRafS5* in several organs from *Vitis vinifera* cv. Vinhão **(C)**. Results indicate the mean ± SD of three independent experiments. Statistical differences between mean values are marked with asterisks (^*^p < .05; ^**^p < .01; ^****^p < .0001).

Because *VviRafS5* was the most expressed raffinose synthase in woody tissues during cold treatment, its transcription was studied in different grapevine organs (canes, leaves, flowers, and roots) and berries at two development stages (green and mature). Results showed that *VviRafS5* is mainly expressed in mature berries, but also in flowers and roots (Figure 6A). Following previous results showing that *RafS* is responsive to different abiotic stresses (Egert et al., 2013) and the identification of several *cis*-regulatory elements involved in abiotic stresses and ABA responses in *VviRafS5* promotor (Figure 6B), cultured cells of cv. Gamay Fréaux were subjected to different temperatures and elicited with salt and ABA to check their effect on the expression of *VviRafS5*. In agreement with previous data, the steady-state transcript levels of *VviRafS5* resulted higher after cold (2.4-fold) and ABA treatments (1.7-fold), and lower (.2-fold) after heat treatment. On the contrary, salt stress did not affect significantly the expression of *VviRafS5* (Figure 6C).

## Discussion

In this study, the accumulation of sugars in grapevine woody tissues in response to cold was explored using an *in vitro* approach to mimic winter cold acclimation. The general accumulation of soluble sugars in grapevine cv. Vinhão in response to cold is in agreement with previous studies. A similar result was obtained in field-grown cv. Riesling and Chardonnay vines (Hamman et al., 1996; Todaro and Dami, 2017). This cold-induced sugar increase most likely comes from starch degradation because a concomitant decrease in this polymeric carbohydrate was observed. This pattern of starch degradation and synthesis of compatible solutes during winter has been also described in the wood of different plants, including poplar (Sauter, 1988; Sauter and van Cleve, 1990), *Cornus sericea* (Ashworth et al., 1993), and willow (Ögren, 1999). Nonetheless, starch degradation may, to some extent, fuel other cellular processes considering the artificial system used in this study, but no raffinose synthesis was observed at room temperature supporting a specific role of RFOs in plant response to cold.

In the present study, we observed that within six raffinose synthase and one stachyose synthase genes identified, *VviRafS5* and *VviRafS8* seem to play pivotal roles during cold treatment of grapevine canes. Furthermore, *VviRafS6*, which was recently associated with grapevine cold response in plantlets (Chai et al., 2019), was also upregulated in woody tissues. To the best of our knowledge, the present study is innovative regarding the characterization of RafS in woody tissues of grapevine. In particular, we confirmed that *VviRafS5*, which was transcriptionally upregulated up to 40-fold in response to cold, is able to synthesize raffinose *in vitro* after its heterologous expression in yeast. Thus, the accumulation of raffinose in grapevine perennial organs by *VviRafS5* may constitute an important metabolic adjustment in response to cold acclimation, alone or in cooperation with other *VviRafS*. Interestingly, despite the observed stachyose accumulation in canes, the expression of *VviStaS1* was not detected in this study, suggesting that in these tissues this RFO is synthesized by a putative RafS, as previously found in *Arabidopsis thaliana* (Gangl and Tenhaken, 2016), or by other still unidentified enzyme.

The results obtained in Gamay Fréaux cultured cells also corroborated the role of *VviRafS5* in cold response. However, in *Arabidopsis thaliana* seeds, the orthologue *AtRafS5* is responsible for raffinose accumulation in response to different stresses, including cold, heat, and drought (Egert et al., 2013; Gangl and Tenhaken, 2016). Therefore, future studies could address the role of RFOs in grapevine response to other abiotic stresses besides cold, including drought and heat, together with the characterization of genes/proteins involved on its biosynthesis. Furthermore, additional studies are still needed to clarify whether *VviRafS8* and *VviRafS6* are indeed grapevine raffinose synthase enzymes because it has been previously shown that several *RafS* from *Arabidopsis thaliana* is not involved in raffinose accumulation (Egert et al., 2013) and *AtSIP2*, previously annotated as a raffinose synthase gene, codes a raffinose-degrading α-galactosidase (Peters et al., 2010). Thus, the characterization of the biochemical activities of these enzymes *in planta* is of the utmost scientific relevance.

The observation in the present work that *VviRafS5* is highly expressed in mature grape berries also deserves further attention because these fruits also contain raffinose (Kliewer, 1964). Given the already established role of RFOs accumulation in response to drought (Egert et al., 2015; Li et al., 2020), it is highly plausible that both raffinose and stachyose, whose synthesis could be mediated by *VviRafS5* and other *RafS*, may have such functions in mature berries.

It is well known that, besides having a central role in seed dormancy, abscission, and abiotic stress signaling, ABA is pivotal in the plant response to low temperature (Eremina et al., 2015). In grapevine buds, the role of ABA during bud dormancy acquisition (Düring and Bachmann, 1975; Koussa et al., 1994; Or et al., 2000) and release (Zheng et al., 2015) is consensual. Although the site of ABA formation and its transport are still a matter of debate (McAdam and Brodribb, 2018), it has recently been reported that phloem companion cells, guard cells, and mesophyll cells are able to synthesize this hormone (Bauer et al., 2013; Kuromori et al., 2014; McAdam and Brodribb, 2018). Remarkably, our results strongly suggested that grapevine woody tissues are able to *de novo* synthesize ABA during cold incubation, which is also an important novelty of the present study. In this regard, not only the levels of ABA and its catabolites were higher during cold acclimation, but also the expression of *VviNCED2* and *− 3* genes was upregulated. *VviNCED2* and *− 3* code for 9-*cis*-epoxycarotenoid dioxygenases and thus are considered major control points of ABA synthesis. Their role in the accumulation of this hormone in grapevine is well known in grapevine buds (Zheng et al., 2015) and plantlets (He et al., 2018), as well as in other species like *Arabidopsis thaliana* (Lefebvre et al., 2006), tobacco (Qin and Zeevaart, 2002), and tomato (Thompson et al., 2000). Taken together, our results suggest that ABA upregulates *VviGolS* and *VviRafS* in grapevine woody tissues during cold acclimation, promoting an accumulation of RFOs, as previously shown in *Boea hygrometrica* (Wang et al., 2009) and alfalfa (Blöchl et al., 2005). Nonetheless, the ABA regulatory network in grapevine woody tissues during cold incubation needs to be further explored, particularly the identification of the transcription factors behind *VviGolS* and *VviRafS* increased expression. Also, and despite that no changes were found in the expression levels of *VviBAMs* (Supplementary Figure 3), the role of the CBF regulon in RFO synthesis should be explored, since it has been shown that *GolS* is a target of these regulatory network (Taji et al., 2002; Tarkowski and Van den Ende, 2015). Furthermore, additional evidence is still necessary to clarify the molecular mechanisms involved in grapevine against cold stress mediated by raffinose and other RFOs.

## Data Availability Statement

The original contributions presented in the study are included in the article/Supplementary Material, and further inquiries can be directed to the corresponding author.

## Author Contributions

HN and HG conceptualized the work and wrote the manuscript. HN, AS, and TS performed laboratory sample processing, biochemical analysis, targeted transcriptomics, and heterologous expression. SF and GD performed metabolomic analysis and data treatment. HN, HG, and GD analyzed the results. AS, TS, GD, HN, SF, and HG reviewed the manuscript. All authors contributed to the article and approved the submitted version.

## Funding

The work was supported by National Funds by FCT—Portuguese Foundation for Science and Technology, under the strategic program UIDB/04050/2020. The work was also supported by FCT and European Funds (FEDER/POCI/COMPETE2020) through the research projects MitiVineDrought (PTDC/BIAFBT/30341/2017 and POCI-01-0145-FEDER-030341), BerryPlastid (PTDC/BIA-FBT/28165/2017 and POCI-01-0145-FEDER-028165), and AgrifoodXXI (NORTE-01-0145-FEDER-000041). AS and HN were supported by FCT grants SFRH/BD/135782/2018 and SFRH/BPD/115518/2016, respectively. This work also benefited from the networking activities within COST ACTION CA17111 INTEGRAPE and Collaborative Laboratory “CoLab ADVID Vines & Wines.”

## Conflict of Interest

The authors declare that the research was conducted in the absence of any commercial or financial relationships that could be construed as a potential conflict of interest.

## Publisher’s Note

All claims expressed in this article are solely those of the authors and do not necessarily represent those of their affiliated organizations, or those of the publisher, the editors and the reviewers. Any product that may be evaluated in this article, or claim that may be made by its manufacturer, is not guaranteed or endorsed by the publisher.

## Footnotes

^1^ http://phytozome.jgi.doe.gov
