# Supplementary material for: VviRafS5 Is a Raffinose Synthase Involved in Cold Acclimation in Grapevine Woody Tissues
Source: Front Plant Sci. 2022 Feb 15;12:754537. doi: 10.3389/fpls.2021.754537 (PMC8885518; doi:10.3389/fpls.2021.754537)
Supplement: Supplementary file 1 [file Data_Sheet_1.docx]

**Supplementary Material**

**Supplementary Table 1**: **–** Primers used in this study.

| qRT-PCR | | | |
| --- | --- | --- | --- |
| Gene | **Primer Forward (5’-3’)** | **Primer Reverse (5’-3’)** | **Accession number (V3)** |
| *VviGolS1*  (Pillet et al. 2012) | TGATTACAGCAGCGTTTTGCC | CGAGAGTACTGGCCTCTTCTAG | Vitvi07g00457 |
| *VviGolS3* | TGCCGAGCAGGACTATTTGA | CAAGGTTGTAGGTGGGTGGA | Vitvi14g02461 |
| *VviRafS1* | TCCTGCCAGAGATGGAATAAGCC | GGCAATTGAACACACCCACGAC | Vitvi19g00768 |
| *VviRafS3* | CCCGCGATGGTATCAGTTTGTTG | TGGCAGTTGTACACTCCAATCAC | Vitvi11g00513 |
| *VviRafS4* | GCAGTTGCAAGAGCATCAGAGG | TCAGCTGTGCTATGGTTGCTATG | Vitvi17g00957 |
| *VviRafS5* | CCATCTTGAGGTGCCAGTACTACG | ATGGTGTTGCCGTCGTGAAGAG | Vitvi12g00076 |
| *VviRafS6*  (Chai et al. 2019) | TCTTCGGATGATGTTGAGT | CAGGAGGGTGATGTTGAT | Vitvi05g00139 |
| *VviRafS8* | GGTGGTGCAGAGAAACACGAAG | CGGGTTTGCCAAGCATGAAACG | Vitvi17g00885 |
| *VviNCED2* (Carbonell-Bejerano et al. 2013) | TGCCTGTCGGTAGAATTGGA | ACACATAAGGGAGGAGACGAAA | Vitvi10g00821 |
| *VviNCED3* (Carbonell-Bejerano et al. 2013) | CCAAACTCTCTACAGTAACTATCC | CTGGTCCCTCAAAACTTTCTC | Vitvi19g01356 |
| *VviBAM2* | CCAGTTTACGTAATGCTCCCTCTG | GCTGTTTCTCAAGCCCGTCTTTG | Vitvi12g00558 |
| *VviBAM3* | GTTCAAGGGTGCCAGTGTTT | GCTTTAGTCCATGCCTTTGC | Vitvi02g00605 |
| *VviBAM4* | TGCAACCCTTGCATTTGAGGAAAC | CACCAATAGACTGCTGGAACCTTG | Vitvi02g01232 |
| *VviBAM5* | CGCCAACCTATCTTTGCAGTCC | TCTTCGACACGGTCCATATCTGC | Vitvi19g00678 |
| *VviACT1*  (Conde et al. 2015) | GTGCCTGCCATGTATGTTGCCATTCAG | GCAAGGTCAAGACGAAGGATAGCATGG | Vitvi04g01613 |
| *VviGAPDH* (Gainza-Cortés et al. 2012) | TTCCGTGTTCCTACTGTTG | CCTCTGACTCCTCCTTGAT | Vitvi17g01598 |
| *VviRafS5* cloning | | | |
| Gene | **VvRafS5-Gap-FW**  **(5’-3’)** | **VvRafS5-Gap-RV**  **(5’-3’)** | **Accession number (V3)** |
| *VviRafS5* | ATATTAAGCTATCAAACAAGTTTGTACAAAATGGCTCCCAGCTTGAGTAA | ACCGAGGAGAGGGTTAGGGATAGGCTTACCGAATAAGTACTCGATCAGGG | Vitvi12g00076 |

**References:**

Carbonell-Bejerano, P., Santa María, E., Torres-Pérez, R., Royo, C., Lijavetzky, D., Bravo, G., et al. (2013) Thermotolerance responses in ripening berries of *Vitis vinifera* L. cv Muscat Hamburg. *Plant Cell Physiol.* 54(7), 1200-1216. doi: 10.1093/pcp/pct071

Pillet, J., Egert, A., Pieri, P., Lecourieux, F., Kappel, C., Charon, J., et al. (2012) VvGOLS1 and VvHsfA2 are involved in the heat stress responses in grapevine berries. *Plant Cell Physiol.* 53(10), 1776-1792. doi: 10.1093/pcp/pcs121

Chai, F., Liu, W., Xiang, Y., Meng, X., Sun, X., Cheng, C., et al. (2019) Comparative metabolic profiling of *Vitis amurensis* and *Vitis vinifera* during cold acclimation. Hortic. Res. 6, 8. doi: 10.1038/s41438-018-0083-5

Conde, A., Regalado, A., Rodrigues, D., Costa, J. M., Blumwald, E., Chaves, M.M., et al. (2015) Polyols in grape berry: transport and metabolic adjustments as a physiological strategy for water stress tolerance in grapevine. *J. Exp. Bot.* 66, 889-906. doi: 10.1093/jxb/eru446

Gainza-Cortés, F., Pérez-Dïaz, R., Pérez-Castro, R., Tapia, J., Casaretto, J. A., González, S., et al. (2012) Characterization of a putative grapevine Zn transporter, VvZIP3, suggests its involvement in early reproductive development in *Vitis vinifera* L. *BMC Plant Biol.* 12, 111. doi: 10.1186/1471-2229-12-111

**Supplementary Figure 1**

**
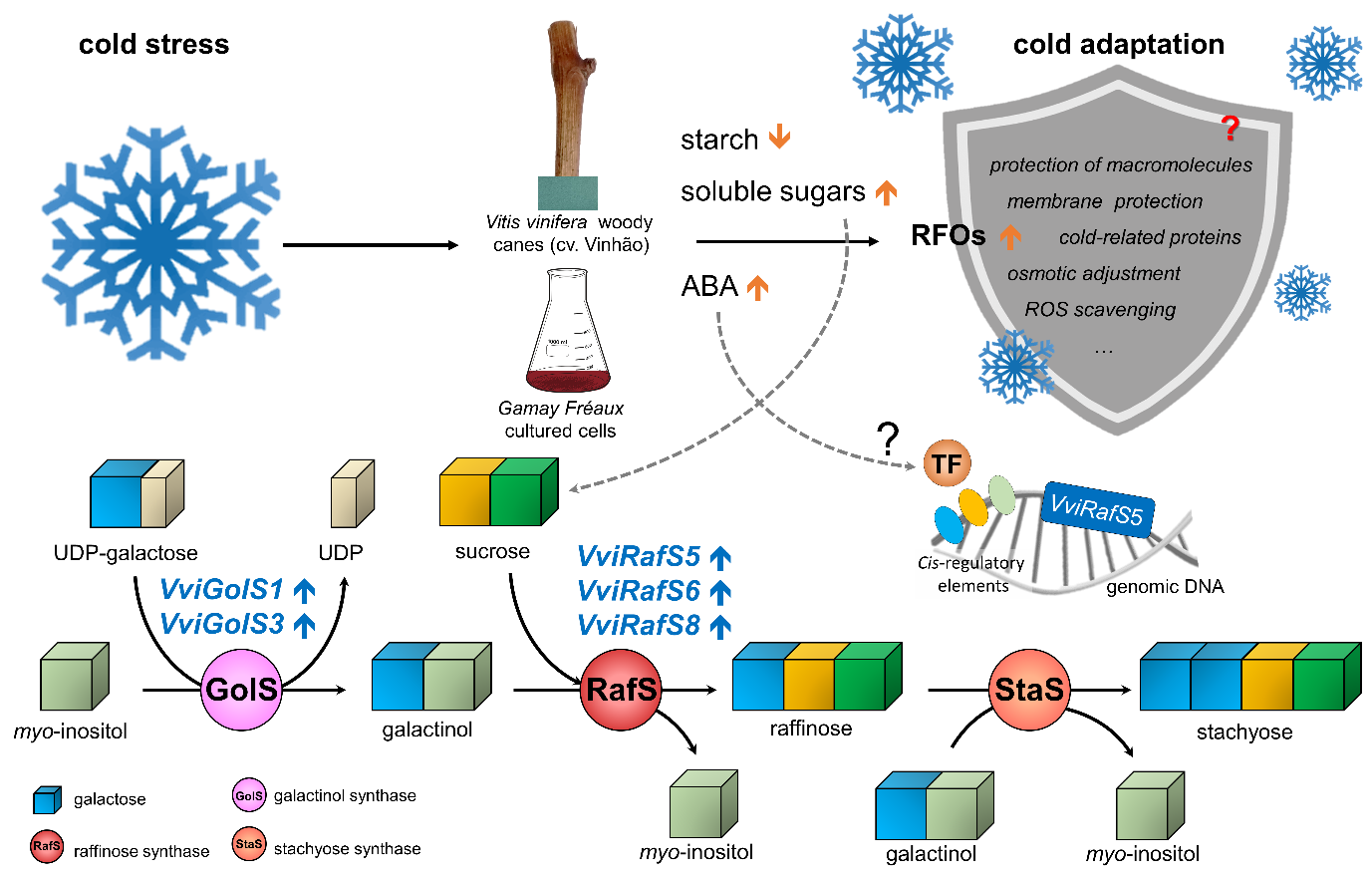
**

**Supplementary Figure 1 –** A proposed model for the biosynthesis of RFOs in grapevine woody tissues during cold acclimatation.

**Supplementary Figure 2**

**
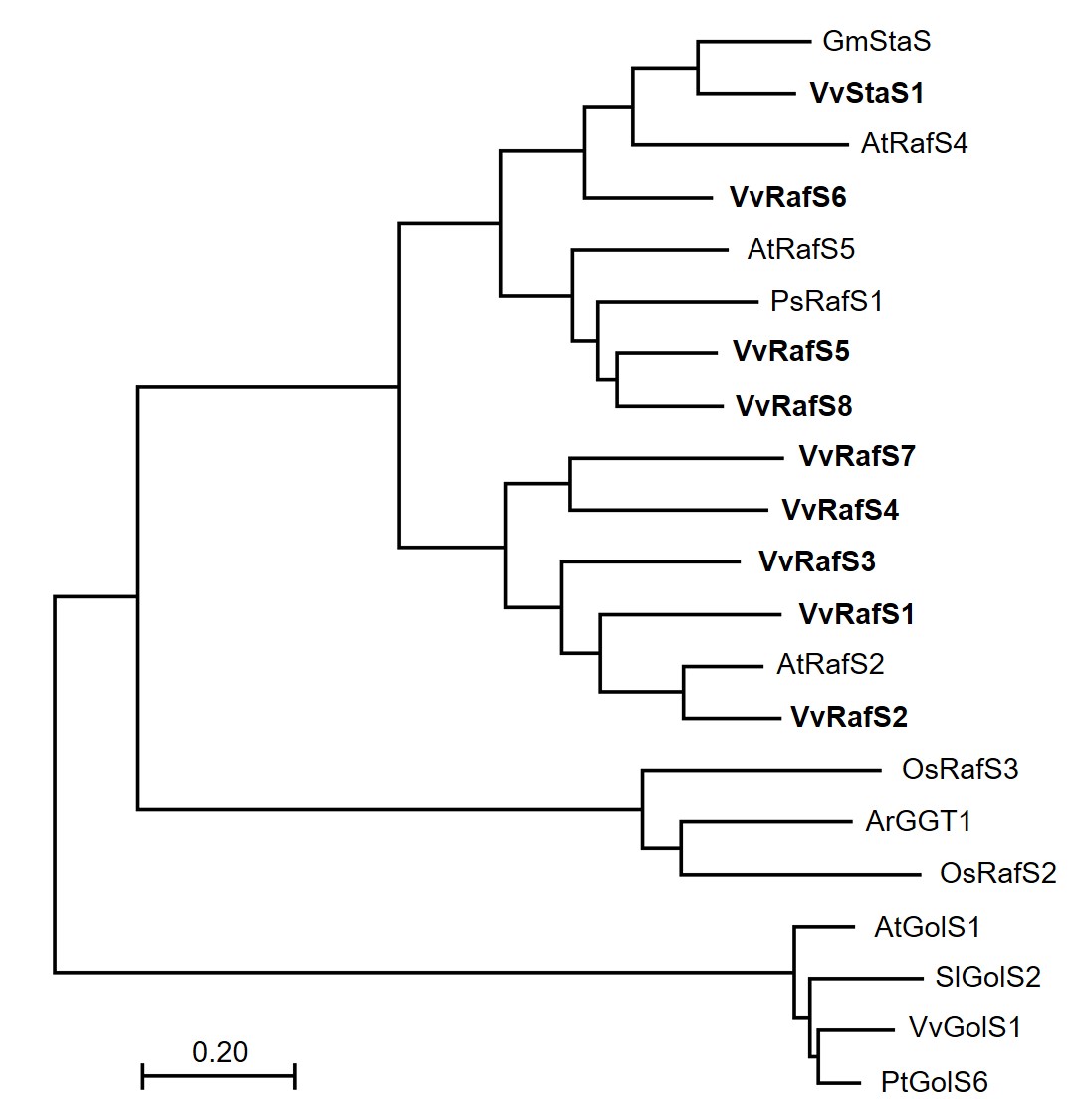
**

**Supplementary Figure 2** – Phylogenetic tree of RafS proteins. Amino acid sequences from Vitis vinifera (see Supplementary Table 1), Arabidopsis thaliana (AtRS2, Q94A08; AtRS4, Q9SYJ4; AtRafS5, Q9FND9; AtGolS1, O22893), Glycine max (GmStaS1, I1NBD9), Ajuga reptans (ArGGT1, AY386246), Populus trichocarpa (PtGolS6, A0A2K1XU51), Pisum sativum (PsRafS1, Q8VWN6), Solanum lycopersicum (SlGolS2, C7G304) and Oryza sativa (OsRafS2, A0A0P0X580; OsRafS3, Q69R81) were aligned using ClustalW method. The tree was constructed using the Neighbor-Joining method and the percentage of replicates in which the proteins clustered together are represented by the bootstrap values near the branches (1000 replicates). The scale bar represents the number of amino acid substitutions per site.

**Supplementary Figure 3**

**
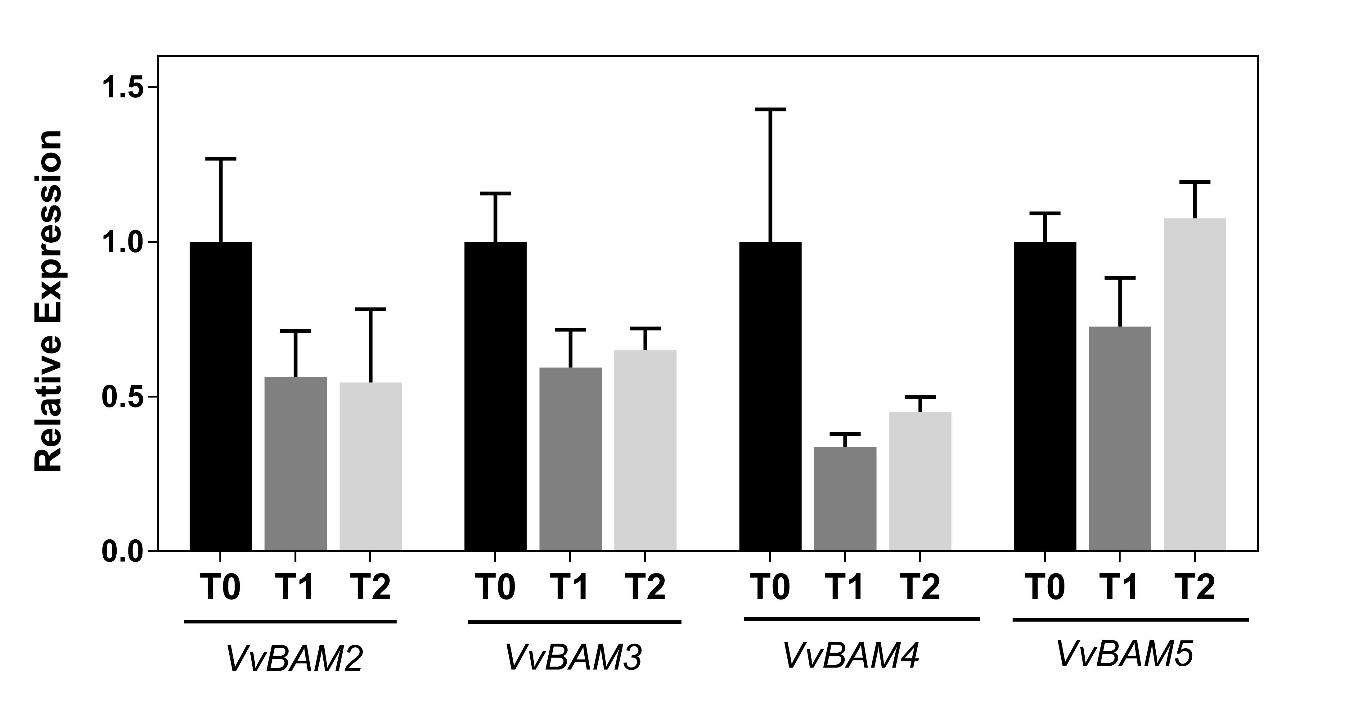
Supplementary Figure 3 -** Transcriptional analysis of *VviBAMs* in *Vitis vinifera* cv. Vinhão canes incubated at 4 ºC up to 14 days. Results indicate the mean ± SD of three biological replicates per condition. Primers used listed in Supplementary Table 1.
